# Why the ongoing occupation of Ukraine matters to ethnobiology

**DOI:** 10.1186/s13002-022-00523-x

**Published:** 2022-03-22

**Authors:** Nataliya Stryamets, Julia Prakofjewa, Giulia Mattalia, Raivo Kalle, Baiba Pruse, Dauro M. Zocchi, Renata Sõukand, Andrea Pieroni, Michele F. Fontefrancesco

**Affiliations:** 1 Department of Environmental Sciences, Informatics and Statistics, Ca’ Foscari University of Venice, Via Torino 155, Mestre, 30172 Venice, Italy; 2 University of Gastronomic Sciences, Piazza Vittorio Emanuele II 9, 12042 Pollenzo, Italy

**Keywords:** Ethnobiology, Biocultural diversity, Ukraine

## Abstract

Ethnobiology and ethnomedicine investigate the continuously changing complex and inextricable relations among culture, nature, and health. Since the emergence of modern ethnobiology a few decades ago, its essence and mission have been the study of biocultural diversities and the centers of its inquiries have been and are local communities and their co-evolutionary interrelationships between natural environments and social systems. At the core of ethnobiologists’ work there are not only conceptualizations of and reflections on others' views about nature and the universe, but also a robust commitment to advocacy in defense of these assemblages of local ecological knowledge, practices, and beliefs (LEK). Homogenization processes and therefore erosion of LEK have occurred throughout history in different ways: from colonialism to industrialization, and from financialization to globalization; however, we cannot forget the role played by centripetal states and even dictatorships in this process, nor the associated political ideology of nationalism, which has often elicited and justified policies aimed at standardizing diversities within state borders. Our research groups have been working since eight years together with local communities in Ukrainian rural areas and documented a remarkable erosion of LEK during the Soviet times, yet an extraordinary surviving biocultural diversity occurs; the ongoing military occupation of Ukraine could further threaten this heritage. While citizens’ attention now should be on effectively supporting those who are experiencing hardships during this traumatic time, ethnobiologists will be called hopefully soon to directly participate in rebuilding the biocultural “cobwebs” damaged by the military operations.

Ethnobiology and ethnomedicine are relatively new disciplines aimed at investigating the continuously changing complex and inextricable relations among culture, nature, and health. Since the emergence of ethnobiology a few decades ago, its essence and mission have been the study of *biocultural diversity* [1]. That is the reason local communities have been and are at the very center of ethnobiological inquiries: local communities “happen” there in a *situated* field, where the very sense of these dynamic relations between nature, humans, and health are articulated, practiced, enjoyed, changed, even disputed. For over a century, ethnobiological works have explored this intimate richness and creative effervescence of local communities, “traditional” societies, and Indigenous Nations, and, since the Conference of Rio in 1992 and the foundation of the International Society of Ethnobiology, ethnobiologists have forged conceptualizations of and reflections on others' views about nature and the universe. Our interest, however, has not been merely speculative; at the core of our work there is a commitment to advocacy in defense of these assemblages of local ecological knowledge, practices, and beliefs (LEK) and their holders [2].

Homogenization processes and therefore erosion of LEK have occurred throughout history in different ways: from colonialism to industrialization, and from financialization to globalization; all of these various phenomena have remarkably contributed to the ongoing depletion of biocultural diversities. However, we cannot forget the role played by centripetal states and even dictatorships in this process, nor the associated political ideology of nationalism, founded on the concept of the state-nation, which has often elicited and justified policies aimed at standardizing all cultural diversities within state borders, leaving space only for the cultural archetype of the national citizen [3–5]. Far from being dissolved by globalization, nationalism, often boosted by religious ideologies, has endured and has generated conflicts and violence, even in recent years [6].

The events that have transpired since February 24, 2022 in Ukraine confirm this and are crucial to the entire ethnobiological community because of their ethical and epistemological implications.

Ukraine has a thousand-year history and diverse cultural traditions and customs, as it is home to numerous minority ethnic groups including Russians, Jews, Belarusians, Moldovans, Greeks, Crimean Tatars, Gagauzes, Bulgarians, Poles, Hungarians, and Romanians. Historically, after the collapse of the Soviet Union independent multiethnic states emerged, confronting state-building and economic transformation crises, with uncertain identities, contested boundaries, and insecure ethnic and/or linguistic minorities.

Some scholars and, in particular, our research groups, during the past eight years, have been working together with local communities in Ukraine to document the trajectories of local ecological knowledge and its changes [7–16], and also attempting to promote its valorization for sustainable development in small scale food chains [17]. We have been able to clearly observe that socioeconomic conditions and political processes during the Soviet period have been the main drivers of the erosion of biocultural diversity: Mountains did not necessarily offer protection against the homogenization policies; yet forests provided shelter from the Holodomor—the artificial hunger (and crime against humanity) imposed on the peasantry in the 1930s. We have visited several villages, even the most remote ones (Fig. 1), and have met hundreds of LEK holders, documenting a rich biocultural diversity, also in informal street markets [18] (Fig. 2).

**Fig. 1:**
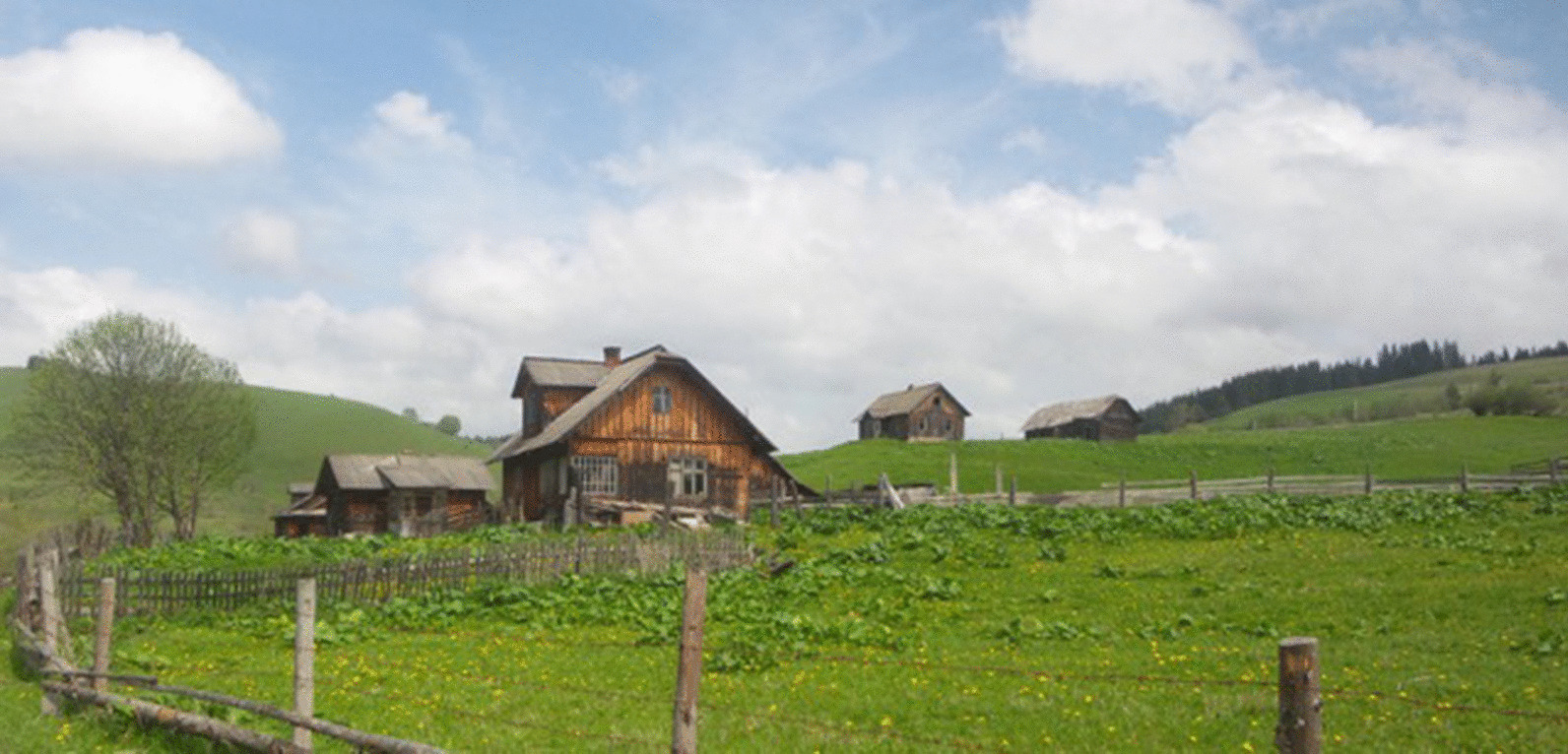
Sarata, one of the Ukrainian most isolated mountain villages in Bukovina (Photo: A Pieroni, May 2015)

**Fig. 2:**
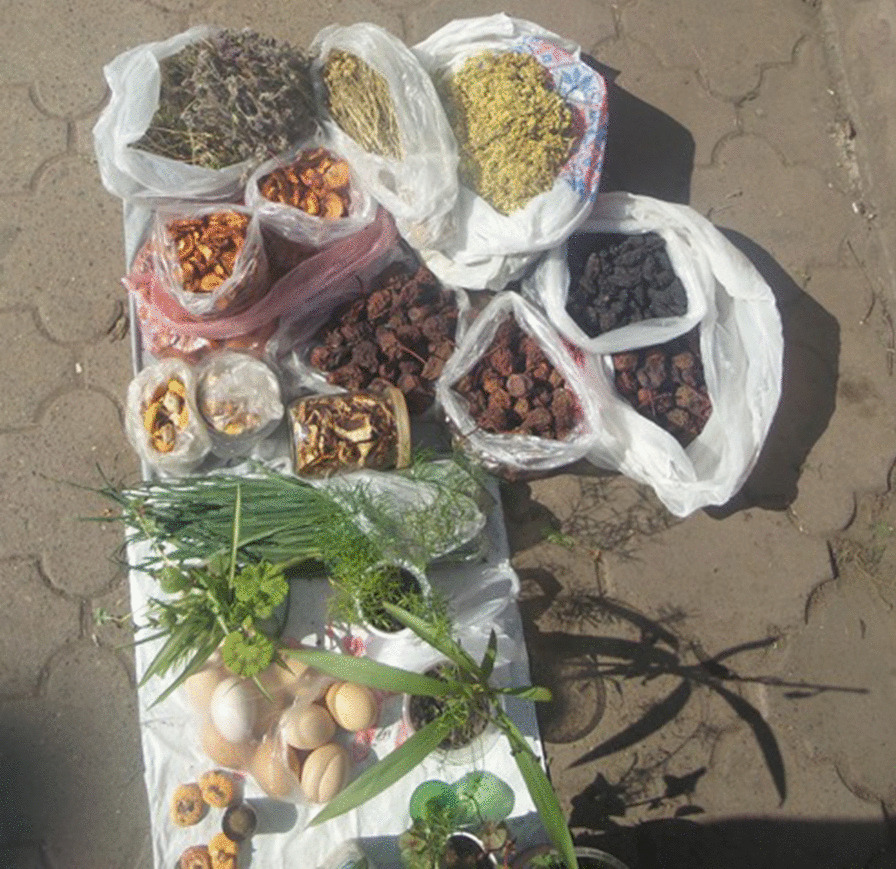
Domestic food products informally sold in a street of Ivano-Frankivsk (Photo: A. Pieroni, May 2016)

Military occupations destroy the pillars of biocultural diversity that distinguish a community, a region, or a country, leaving behind an expanse of rubble weakened in its uniqueness. Forcing local communities to adopt another's language and culture is the first step of culturicides (systematic destruction of cultures) and tears apart the social connection with the local biodiversity, causing its erosion (in both the short and long term, also considering the traumatic experience of war). “Strangers” cannot normally care about the local environment, and thus are not able to understand the intimate relations and values local communities have created during their longstanding exchange with the surrounding nature [19].

War is a border experience, and its very essence is cultural polarization and therefore, it is essential to maintain the world's complexity, multidimensionality, ambiguity, and diversity. Now citizens’ attention is on effectively supporting those who are experiencing hardships during this traumatic time. Tomorrow, however, ethnobiologists will be called upon even more to be together with local communities, to celebrate their folk knowledge and expertise, and to directly participate in rebuilding the biocultural “cobwebs” damaged by the military operations.

## References

[1] Maffi L (2001). On biocultural diversity: linking language, knowledge, and the environment.

[2] International Society of Ethnobiology. Mission. https://www.ethnobiology.net/what-we-do/. Accessed 12 March 2022.

[3] Gellner E (2006). Nations and nationalism.

[4] Anderson B (1983). Imagined communities.

[5] Hastings A (1997). The construction of nationhood: ethnicity, religion and nationhood.

[6] Mylonas H, Tudor M (2021). Nationalism: what we know and what we still need to know. Ann Rev Pol Sci.

[7] Elbakidze M, Angelstam P (2007). Implementing sustainable forest management in Ukraine's Carpathian Mountains: the role of traditional village systems. For Ecol Manag.

[8] Melnykovych M, Nijnik M, Soloviy I, Nijnik A, Sarkki S, Bihun Y (2018). Social-ecological innovation in remote mountain areas: adaptive responses of forest-dependent communities to the challenges of a changing world. Sci Total Environ.

[9] Sõukand R, Pieroni A (2016). The importance of a border: medical, veterinary, and wild food ethnobotany of the Hutsuls living on the Romanian and Ukrainian sides of Bukovina. J Ethnopharmacol.

[10] Pelyukh O, Lavnyy V, Paletto A, Troxler D (2021). Stakeholder analysis in sustainable forest management: an application in the Yavoriv region (Ukraine). For Pol Econ.

[11] Pieroni A, Sõukand R (2017). Are borders more important than geographical distance? The wild food ethnobotany of the Boykos and its overlap with that of the Bukovinian Hutsuls in Western Ukraine. J Ethnobiol.

[12] Pieroni A, Sõukand R (2018). Forest as stronghold of local ecological practice: currently used wild food plants in Polesia. Northern Ukraine Econ Bot.

[13] Mattalia G, Stryamets N, Pieroni A, Sõukand R (2020). Knowledge transmission patterns at the border: ethnobotany of Hutsuls living in the Carpathian Mountains of Bukovina (SW Ukraine and NE Romania). J Ethnobiol Ethnomed.

[14] Stryamets N, Mattalia G, Pieroni A, Khomyn I, Sõukand R (2021). Dining tables divided by a border: the effect of socio-political scenarios on local ecological knowledge of Romanians living in Ukrainian and Romanian Bukovina. Foods.

[15] Mattalia G, Stryamets N, Balázsi Á, Molnár G, Gliga A, Pieroni A, Sõukand R, Reyes-García V (2021). Hutsuls’ perceptions of forests and uses of forest resource in Ukrainian and Romanian Bukovina. Int For Res.

[16] Mattalia G, Stryamets N, Grygorovych A, Pieroni A, Sõukand R (2021). Borders as crossroads: the diverging routes of herbal knowledge of Romanians living on the Romanian and Ukrainian Sides of Bukovina. Front Pharmacol.

[17] Zocchi DM, Motuzenko O, Stryamets N, Fontefrancesco MF, Sõukand R, Pieroni A. Atlas of the Ark of Taste Products of Ukraine. Bra, Italy: University of Gastronomic Sciences and Slow Food (in press).

[18] Sõukand R, Stryamets N, Fontefrancesco MF, Pieroni A (2020). The importance of tolerating interstices: Babushka markets in Ukraine and Eastern Europe and their role in maintaining local food knowledge and diversity. Heliyon.

[19] Lotman YM (2020). Universe of the mind: a semiotic theory of culture.

